# Strengthening the Gluteus Medius Using Various Bodyweight and Resistance Exercises

**DOI:** 10.1519/SSC.0000000000000221

**Published:** 2016-06-03

**Authors:** Petr Stastny, James J. Tufano, Artur Golas, Miroslav Petr

**Affiliations:** 1 Department of Sport, Faculty of Physical Education and Sport, Charles University in Prague, Prague, Czech Republic;; 2 Department of Exercise and Health Sciences, Edith Cowan University, Joondalup, WA, Australia;; 3 Department of Sports Training, The Jerzy Kukuczka Academy of Physical Education, Katowice, Poland; and; 4 Department of Physiology, Faculty of Physical Education and Sport, Charles University in Prague, Prague, Czech Republic

**Keywords:** gluteus medius, work-out program, exercise selection, muscle activity, postactivation potentiation, hip abduction

## Abstract

THE GLUTEUS MEDIUS (Gmed) IS AN IMPORTANT MUSCLE AND, IF WEAK, CAN CAUSE KNEE, HIP, OR LOWER-BACK PATHOLOGIES. THIS ARTICLE REVIEWS METHODS OF Gmed STRENGTH ASSESSMENT, PROVIDES EXERCISES THAT TARGET THE Gmed BASED ON ELECTROMYOGRAPHY, PRESENTS HOW TO IMPLEMENT Gmed STRENGTHENING IN HEAVY RESISTANCE TRAINING PROGRAMS, AND EXPLAINS THE IMPORTANCE OF INCLUDING THESE EXERCISES IN THESE PROGRAMS.

## Abstract 2

Supplemental Digital Content is Available in the Text.

## INTRODUCTION

The process of individualizing workouts is important when designing a strength training program, with exercise selection being of paramount importance. Selected exercises must then be harmonized with other parameters such as exercise intensity, number of repetitions, speed of contraction, rest intervals, and training history to formulate an organized resistance training program. Specifically, exercise selection addresses which muscles in the kinetic chain should be developed to achieve the appropriate kinematics of an exercise or movement. Subsequently, an efficient movement pattern can maintain optimal kinematics up until the point where the weakest muscle cannot maintain, or contribute to, the summation of forces. Therefore, it may be useful to utilize exercises that target weakened, or potentially the weakest, muscle groups within the kinetic chain so that these muscle groups do not limit force production and velocity in multijoint movements during competition.

One example of a weak muscle group's ability to disrupt movement is weakness of the gluteus medius (Gmed), which may result in adverse changes in kinematics (15), an increased risk of injury in athletes (25), and decreased sport performance (28). In support of this, it has been shown that athletes with stronger hip abduction (HAB) strength are less likely to be injured compared with athletes with weaker HAB (25). Furthermore, Gmed injury in competitive sport has been associated with unilateral weakness, rather than a bilateral deficit (47,48). Therefore, the importance of unilateral Gmed strengthening has been largely discussed, and a large number of exercises that target the Gmed have received attention from researchers and practitioners (5,34,35). Recommendations for Gmed strengthening have been made previously, seeming to originate from a rehabilitative standpoint based primarily on anatomical function (34). However, the implication of specific Gmed exercises during heavy resistance training programs has not been clearly summarized. It may be advised to include evidence-based high-intensity Gmed strengthening during heavy resistance training in athletes, as a means of preventing injury and avoiding the need for formal rehabilitation. Therefore, this article focuses on testing methods for determining Gmed weakness in apparently healthy athletes that mitigate the ceiling effect of traditional testing. Additionally, this article summarizes the exercises that result in the greatest muscle activity of the Gmed and explains the importance of including these exercises in a heavy resistance training program.

## QUANTIFYING HIP ABDUCTION STRENGTH

Specific Gmed strengthening is usually based on the knowledge of Gmed weakness, assessed by measuring HAB strength. Although the Trendelenburg test (TT) is commonly used to determine whether Gmed weakness exists in clinical settings, it has been shown to be a poor predictor for Gmed weakness in people without a diagnosed pathology or lower-back pain. Thus, the TT has limited use as a measure of hip abductor function and strength in athletic, nonsymptomatic populations (23,50). Other HAB strength tests can be performed in a supine or side-lying position using an ordinal scale of 0–5 (20). For these tests, the tester can apply resistance to the lateral aspect of the knee (20), and if the tester determines that the patient's strength reaches a subjective level 4 of 5 (34), it is reported that the patient can sufficiently resist against low external forces. However, a subjective scale ranging from 0 to 5 for such “functional” tests does not allow for detailed assessments of healthy resistance-trained athletes, because it is likely that the athlete can achieve the highest possible score during the test but may have relative muscle weakness during competition. Fortunately, HAB tests can be performed with a handheld dynamometer in a supine (2) or side-lying (48) body position, allowing force output to be quantified and expressed in standard units of Newton (N) or Newton meters (N·m) and also pounds (lbs). It is also possible to measure HAB strength through isokinetic dynamometry with specific speed conditions. However, this method of evaluation is possible only in a laboratory setting with trained personnel and specialized equipment. Therefore, assessing HAB strength with a handheld dynamometer may be the most practical method for determining Gmed strength in athletes.

When standardized, handheld dynamometry seems to be more appropriate for athletes compared with the TT because HAB strength can be compared to normative values (2) and individual strength ratios can be quantified. When determining the HAB strength of an athlete, it has been suggested that a bilateral HAB strength deficit of more than 10% is considered to be the clinical milestone that must be reached before returning an athlete to competition after sustaining an injury and completing rehabilitation (46). Additionally, achieving a HAB:adduction ratio of more than 90% (hip adductors are at least 90% of abductors) has been recommended before returning to sport after a hip adductor strain (31). However, it is important to note that these recommendations are general guidelines that may not be appropriate in all situations. Moreover, these data are related to adductor weakness and may not be comparable when HAB weakness or injury is present. Nevertheless, measuring hip strength in the frontal plane using handheld dynamometry makes it possible to determine whether strength deficits are present based on identifying percentages, which is more accurate than a traditional ordinal scale.

If handheld dynamometry is used, it is important to acknowledge that a more detailed procedure may be required. Variables such as body position, type of the test, dynamometer position, and the type of maximum voluntary contraction time must be considered when performing HAB strength measurements. The 2 main tests that can be chosen include the “make test” and the “break test.” A make test can be described as an isometric test during which the athlete is instructed to exert maximum voluntary force against a fixed dynamometer. With the dynamometer placed above the knee, the “make test” uses a relatively short lever that includes only the femur length. Therefore, this method is resulting in a highly standardized isometric condition, which is conducive of measuring maximal force. During the break test, the athlete is also instructed to exert maximum voluntary force against the dynamometer. However, in contrast with the make test, the tester must apply force to the dynamometer to overcome the athlete's force, resulting in an eccentric muscle action. During the break test, the tested limb interacts with external forces (the force applied by the tester), which may be more similar to the conditions that an athlete experiences during competition. Because the break test requires the tester to overcome athlete's strength, the tester must have a mechanical advantage during this test. Therefore, it is advised to place the dynamometer just above the lateral malleolus to create a longer lever, which favors the downward force of the tester. Documentation of testing position and procedure is always recommended so that accurate comparisons are made. This is particularly important when testing positions vary with respect to lever arm (e.g., lateral knee versus ankle).

**Figure 1:**
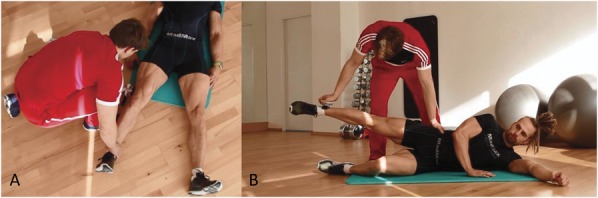
Body and dynamometer position for hip abduction strength measurement using the make test in a supine position (A) and the break test in a side-lying position (B).

If the aim of the test is to assess whether a bilateral HAB strength deficit is present, the athlete should be measured in a supine position using an isometric “make” test (2) (see Video 1, Supplemental Digital Content, http://links.lww.com/SCJ/A187 or Figure 1A) because a side-lying position results in more measurement variation (46). This occurs probably because the side-lying position allows for less stability and bilateral Gmed force production: one side against the table or ground and the other against the dynamometer. Therefore, a supine measurement allows for unilateral strength assessment of each leg independently, without contralateral interference. However, the HAB:adduction strength ratio may be tested in the side-lying position using the “break” test method (48) (see Video 2, Supplemental Digital Content, http://links.lww.com/SCJ/A188 and Figure 1B). Because of the increased ability to produce force in a side-lying position, this method may be most appropriate for measuring an athlete's peak force production. When using such methods, the handheld dynamometer should be placed on the lateral side of the leg, either just above the lateral malleolus of the ankle for the break test or the lateral epicondyle of the knee for the make test, standardizing the length of the lever arm (femur length or leg length). Current recommendations state that a HAB testing protocol should include at least 3 attempts of a 5-s contraction for the make test and 30-s rest between attempts (46); a break test should include at least 2 attempts (48).

## Gmed ACTIVITY ESTIMATION

Based on the relationship between large degrees of muscle activation and subsequent increases in strength (17–19), an initial part of designing a Gmed-specific resistance training program should be selecting exercises that encourage large amounts of Gmed electromyography (EMG) activity. Some practitioners may claim that they can identify muscle activity during exercise because of anatomical position, observational muscle feedback, delayed onset muscle soreness, and increased muscle volume (16,33). However, it is important to know that an individual cannot describe muscle activity without the use of specialized equipment because muscle activity is a multifactorial phenomenon that should only be quantified using EMG.

Because of differences in muscle activation between individuals, surface EMG must be normalized to an individual's maximum standard, which is most often a maximum voluntary isometric contraction (MVIC). Typically, a Gmed MVIC is performed in a side-lying position, with the active lower limb abducted 10–30° from the neutral anatomical position (4,9). However, some studies use other methods to assess MVIC (4), and dynamic exercises have different activation patterns than MVICs (4), making it difficult to compare EMG data during complex resistance exercises, especially the relative muscle activity which varies as the exercise load is increased (30,42).

Although many practitioners associate increases in muscle activity with increases in muscular strength, simply measuring the amount of muscle activation is not sufficient, in itself, to prescribe exercises during a strength training program. It is important to note that variations in the strength ratio between muscle groups can affect muscle activity during an exercise. For example, if HAB strength is greater than knee flexion strength, Gmed activity during the Farmer's walk exercise (walking while carrying dumbbells in the hands at the side of the body) is greater than if knee flexion is stronger than HAB (43). Similarly, it has been shown that during split squats and walking lunges, EMG activity of the quadriceps and hamstrings can differ depending on HAB and knee flexion strength. Therefore, although a strength and conditioning professional can base an evidence-based Gmed-specific training program on exercises that result in large degrees of muscle activity, it is important to understand that muscle activity is not always consistent between athletes across all exercises and that EMG can be used as a starting point when selecting exercises. Hence, the presence of interindividual differences in strength and muscle activation highlights the need for individualized training programs.

## Gmed EXERCISES

Despite the relationship between EMG activity and strength (17,19), the volume of data revealing the amount of Gmed activation during complex, heavily loaded lower-limb exercises is surprisingly low, even in common exercises such as the bilateral squat. A summary of relevant Gmed EMG research is provided in Tables 1 and 2. Table 1 includes compound, multijoint exercises that are often heavily loaded, whereas Table 2 includes accessory exercises, mainly of bodyweight and single-joint nature. Together, these tables provide practitioners a choice of exercises that range in Gmed activity from a high level of activation (41–60% MVIC) to a very high level of activation (>60% MVIC) (35).

**Table 1:**
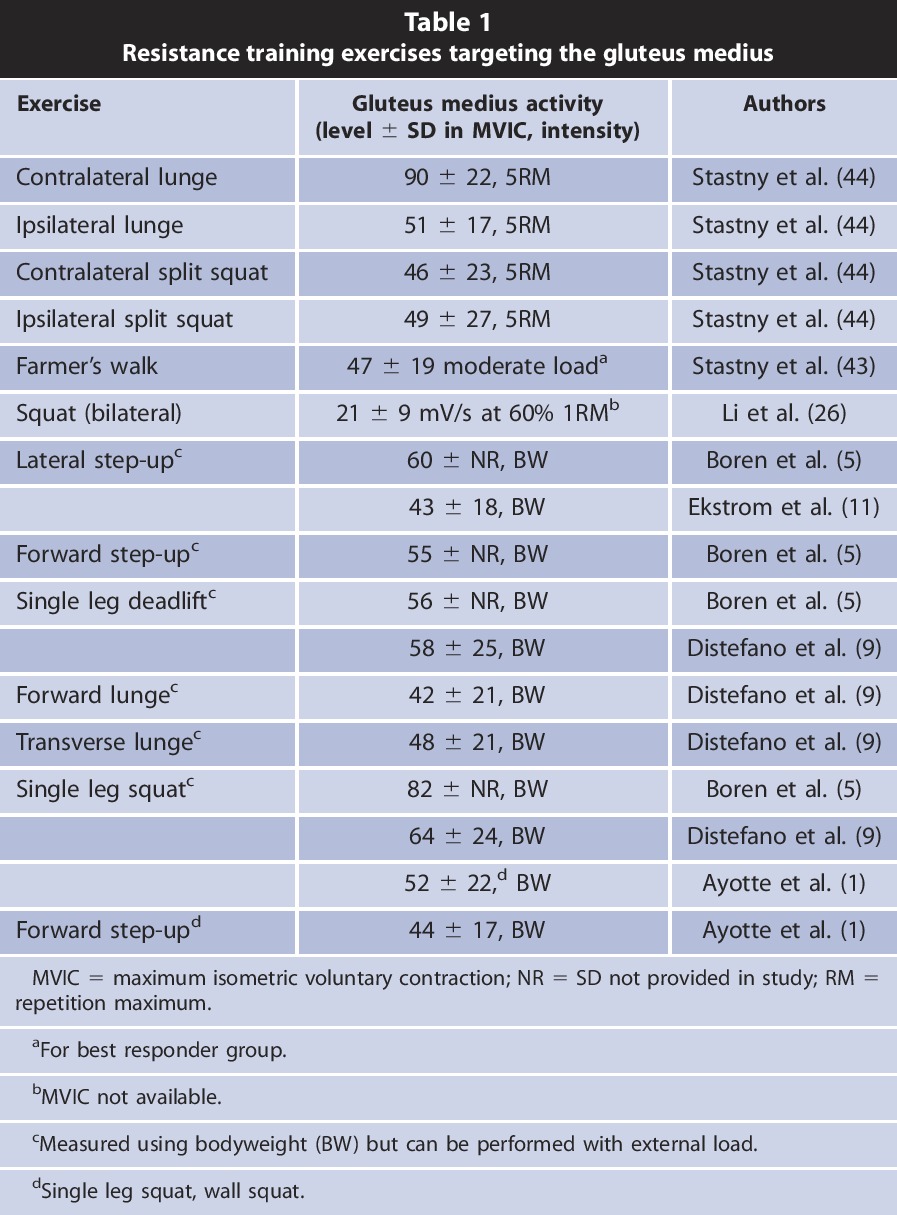
Resistance training exercises targeting the gluteus medius

**Table 2:**
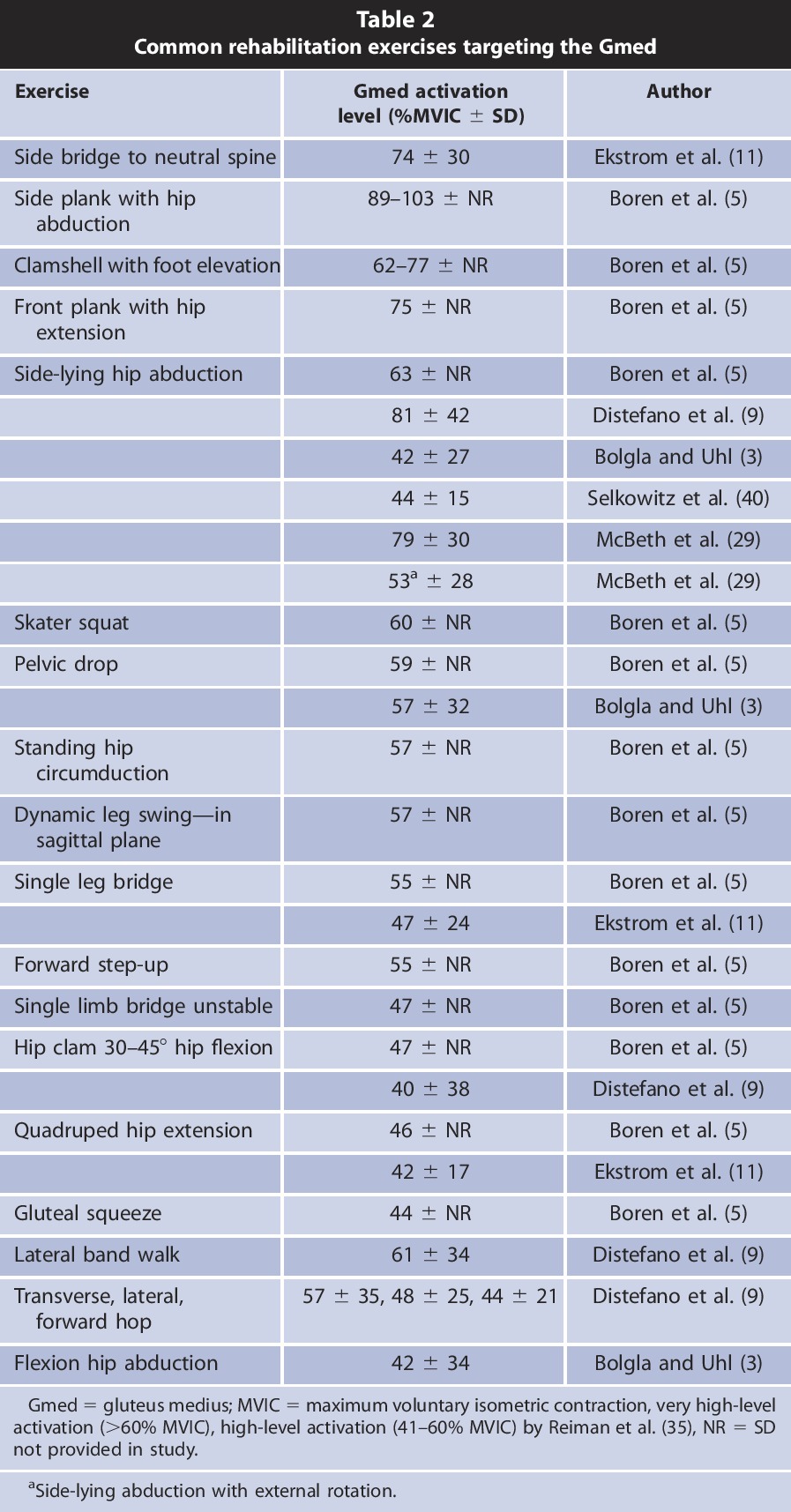
Common rehabilitation exercises targeting the Gmed

Complex exercises such as squats, deadlifts, and step-ups can be heavily loaded, making them preferable compared to single-joint rehabilitation exercises in athletic populations because of their ability to progressively increase exercise intensity, increase the hormonal response (24), and result in satellite cell proliferation (22,49). Therefore, we suggest that heavy resistance exercises (Table 1) may be more effective at inducing functional strength gains of the Gmed in athletes because the force required to overcome external loads may be closer to the force required by an athlete during competition than the force required during unloaded, single-joint exercises.

## PROGRAM DESIGN

When designing a resistance training program, the strength and conditioning professional should target specific aims, which may include the correction of muscle imbalances, increasing performance, or both. Specifically, when choosing Gmed exercises for a resistance training program, the individual needs of sportsmen must be addressed and decisions must be made regarding the use of complex or isolated movements. As previously mentioned, the Gmed training recommendations in this article are primarily designed with healthy athletes in mind, and we suggest that if a pathology is present, or if there is a need for rehabilitation, heavily loaded exercise may need to cease and the training recommendations set forth by Presswood et al. (34) should be considered.

Gmed strengthening becomes increasingly important in an applied sports setting because unilateral HAB weakness has been associated with an increased risk of injury in sports such as soccer, ice hockey, and running (7,14,46,48). Furthermore, Gmed strength may be even more important in sports when the center of mass changes direction unexpectedly, requiring strength and stabilization during unilateral stance. Because of the nature of contact sports and the role of pelvic stability to maintain the summation of forces of movements that begin in the lower extremity, Gmed strengthening should be included in sports that require unilateral support, especially during body-to-body contact. In these sports, unilateral Gmed strengthening while standing can be considered as sport specific. For example, single leg squats with external resistance can be included during the preseason or in-season for ice-hockey players but should not be a staple of an ice-hockey player's general strength development. Some may take this idea further and prescribe such exercises on an unstable surface in an attempt to mimic the instability experienced during competition. Although the Gmed functions as a pelvis and knee stabilizer, doing exercises on an unstable surface does not result in additional activation of the Gmed during squatting (26). Thus, the application of resistance training on unstable surfaces is unwarranted, as it may not effectively increase Gmed activity.

Heavy resistance training differs from rehabilitation in that it aims to evoke a combination of metabolic, endocrine, and neuromuscular responses, often requiring exercise load to be progressively increased up to the repetition maximum (RM). When executing a resistance training program, exercises can be implemented in a variety of ways including traditional sets, agonist-antagonist supersets, and postactivation potentiation (PAP) (work) complexes. This article briefly elaborates on these 3 strategies, laying the foundation for Gmed-specific exercise implementation and providing an overview of how to create effective Gmed-specific training programs to resolve asymmetrical, unilateral Gmed weakness.

### TRADITIONAL TRAINING

A traditional resistance training program that focuses on maximal strength development should include between 3 and 5 sets of an exercise, depending on performance level (41). This recommendation can also be applied to Gmed-specific training. As with most types of resistance training, it is important to include heavily loaded complex exercises (Table 1) at the beginning of a Gmed-specific training session, and less complex, bodyweight exercises (Table 2) can make up the remainder (Table 3).

**Table 3:**
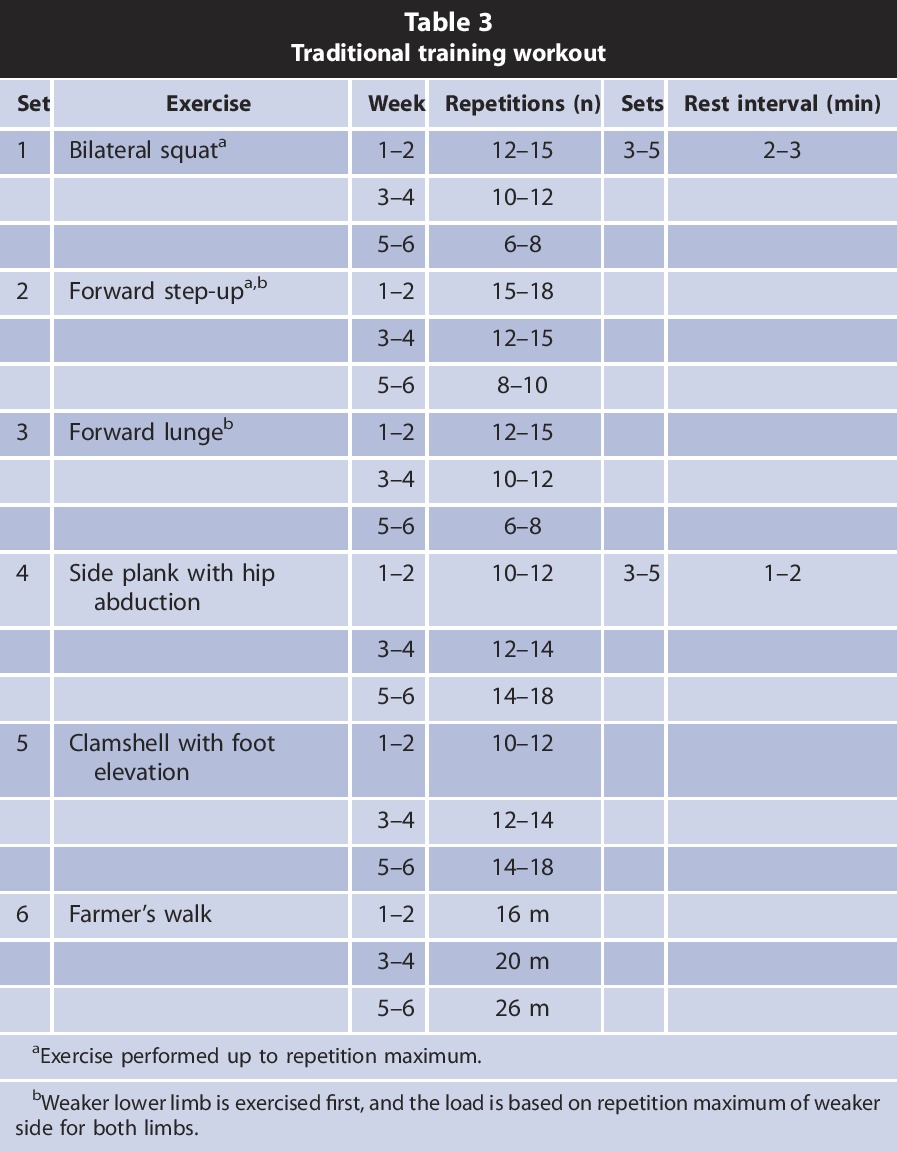
Traditional training workout

After a period of detraining, Gmed exercises should not be performed based on a load representative of a repetition maximum, but instead should be chosen to achieve a subjective rating of perceived exertion with 1–2 minutes of rest. Once accustomed to these loads, exercises can then be performed using RM loads and should be followed by 3–5 minutes rest intervals.

If there is unilateral imbalance in HAB strength, the load and repetitions performed in a training session should be based on the abilities of the weaker side, and the weaker side should be trained first. When HAB imbalances are present, unilateral exercises may be preferred over bilateral ones. Because the functions of the Gmed include abducting the hip; preventing adduction and medial rotation of the femur during complex lateral stabilization of the pelvis; supporting the hip and knee during a single leg stance; and preventing the pelvis from dropping on the opposite side during unilateral stance, most unilateral exercises require large amounts of Gmed activation.

The traditional approach to resistance training suggests that the prime movers should be trained and separately exhausted by combining isolated and complex exercises, which differs from advanced training strategies described below. To increase an athlete's strength, external load should be progressively increased from one workout to another while progressively decreasing the number of repetitions and increasing the number of sets. If exercises are performed that do not allow the external load to be increased, the number of repetitions should be increased to evoke muscle exhaustion. To strengthen the Gmed, we recommend this traditional approach for athletes who are not experienced in resistance training or have been participating in resistance training for less than 3 years to increase their base level of strength before participating in more advanced training strategies.

### AGONIST-ANTAGONIST SUPERSET TRAINING

Strength training using agonist-antagonist supersets allows for short rest intervals to be used without increasing neuromuscular fatigue (36) and can be implemented with complex exercises for experienced athletes as shown in Table 4. The pair of opposing exercises should be coupled using reversed force vectors to primarily target antagonist muscle groups (i.e., overhead presses and pull-ups). In the case of Gmed-specific training, choosing agonist-antagonist movement patterns with opposing force vectors can be challenging, but it is possible. For example, coupling barbell squats with reverse sit ups requires the Gmed to actively extend and externally rotate the hips during squatting and allows the Gmed to relax while hip flexion occurs during reverse sit ups. Although barbell squats may partly fatigue the trunk musculature, the fatigue is mainly experienced in the posterior trunk muscles, opposite of the neuromuscular involvement of reverse sit ups. Agonist-antagonist supersets can also be used by less experienced athletes; but in this case, we would recommend only 2 sets per exercise, as exercise technique may worsen as fatigue accumulates. Because this kind of exercise routine increases exercise volume, we recommend to not exceed 2 sessions per week.

**Table 4:**
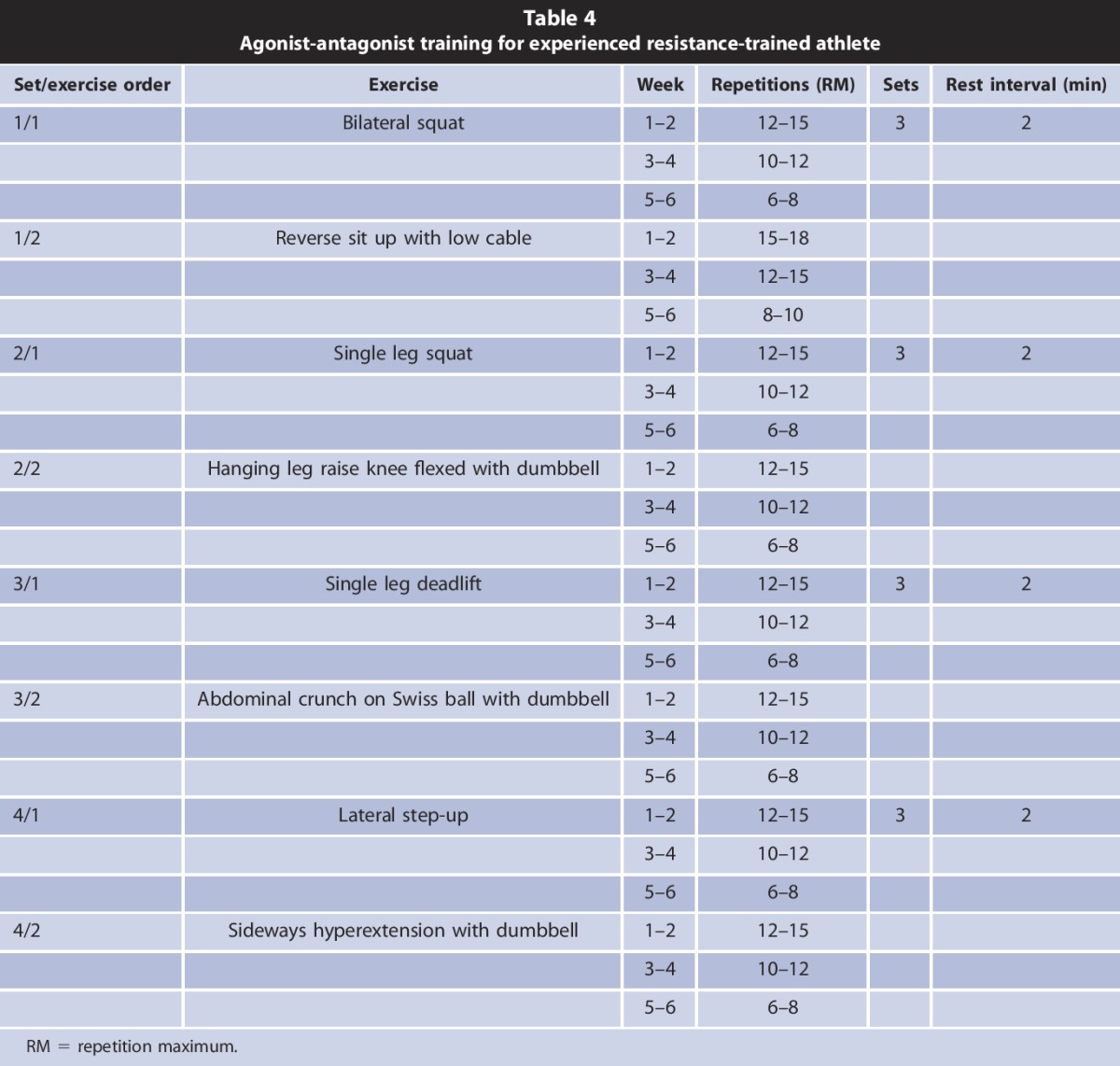
Agonist-antagonist training for experienced resistance-trained athlete

### RESISTANCE TRAINING WITH POSTACTIVATION POTENTIATION

Postactivation potentiation is an advanced training strategy that consists of a conditioning activity aimed at increasing motor unit activation of selected muscles, priming them for a subsequent performance task. It is believed that this phenomenon occurs partly because of increases in low-frequency tetanic force during movement (exercise) after a “conditioning” contractile activity (37). When creating a resistance training session using PAP, there are several factors to consider when implementing the conditioning activity such as the athlete's strength level, the conditioning exercise, and the rest interval between the conditioning activity and the performance task.

In practice, PAP is accomplished by completing a heavily loaded conditioning activity followed by a lighter, more explosive activity. For example, it has been shown that PAP occurs after back squats and power cleans, but the magnitude of improvement during a subsequent sprint performance was greater after power cleans indicating that explosive conditioning activities may be optimal (39). It is recommended that the rest interval between a conditioning activity and the subsequent exercise is between 3 and 7 minutes (13,37,51) when a conditioning activity includes an MVIC for at least 10 seconds or when a 5RM load is used during the conditioning activity (37,51). Because the time course of PAP is very sensitive and varies between athletes, exercises, and exercise sessions (37), the optimal rest interval and number of repetitions used should be discovered using trial and error. Along these lines, it has been shown that PAP is expressed sooner after a less fatiguing conditioning activity compared with a more fatiguing one (6), and the conditioning acitivty's range of motion plays a role in subsequent performance (12,38). It has been shown that PAP is strongly related to strength level, meaning that stronger athletes potentiate quicker than weaker athletes after a conditioning activity (38).

Despite the plethora of research on PAP, the effect of PAP during a Gmed-focused resistance training session remains unknown. However, the recommendations shown in Table 5 are based on previous research. Furthermore, we would recommend that PAP may be effective for 8 weeks, while progressively increasing the number of sets, but that the program may not be as effective after 12 weeks (37).

**Table 5:**
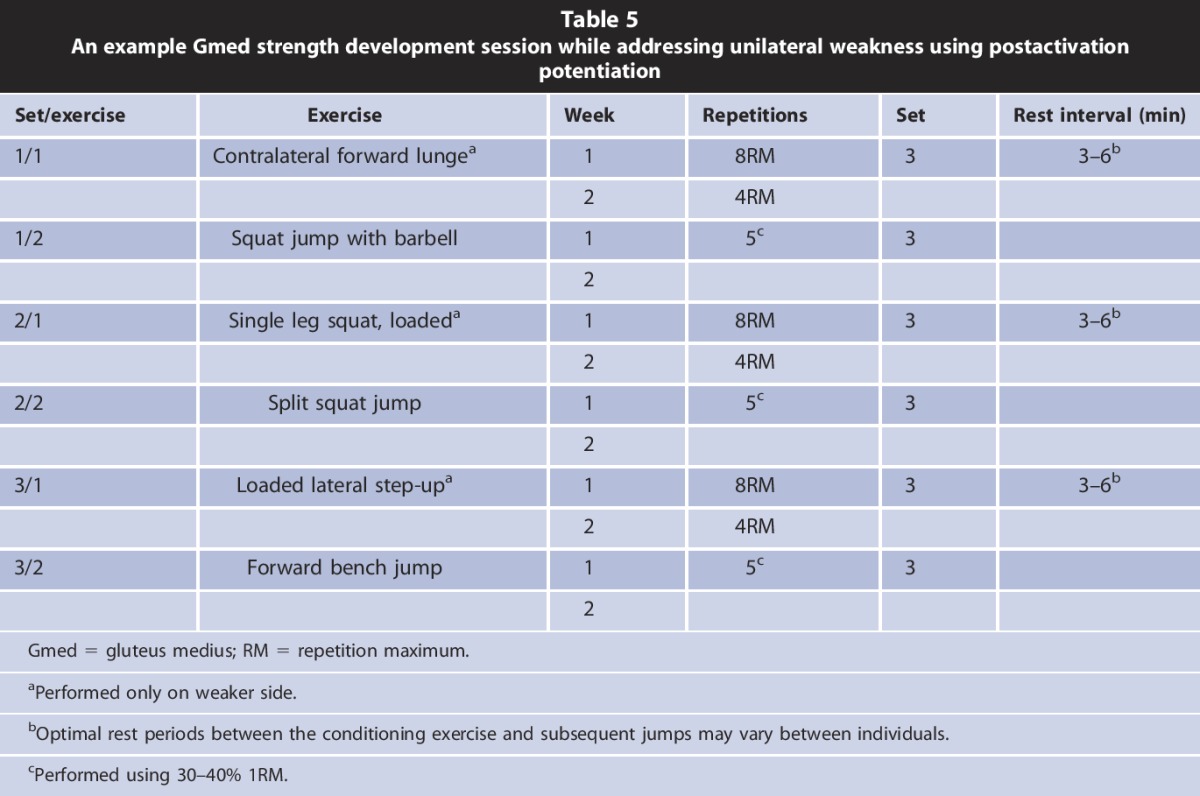
An example Gmed strength development session while addressing unilateral weakness using postactivation potentiation

**Figure 2:**
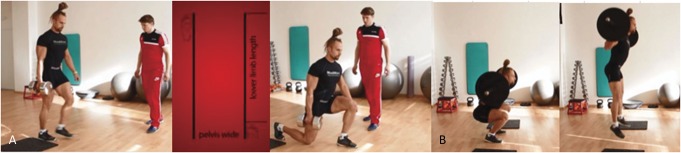
Superset of contralateral forward lunges (A) and squat jumps (B).

**Figure 3:**
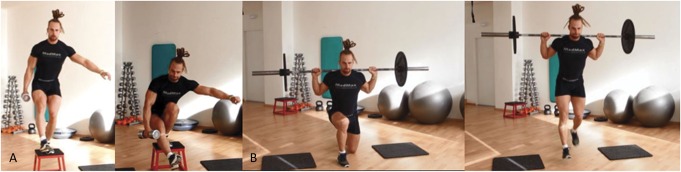
Superset of single leg squats (A) and split squat jumps (B).

It has been reported that a set of forward lunges with a 5RM load held in the contralateral hand in relation to the forward working leg results in greater Gmed than quadriceps or hamstrings activity (44) and thus can be used as a unilateral conditioning activity for the Gmed before bilateral squats (Table 5). After a 3-minute rest, the squat jump is performed as shown in Supplemental Digital Content (see Video 3, http://links.lww.com/SCJ/A189) and Figure 2. In the same manner, the single leg squat can be used as a unilateral conditioning activity for the Gmed before split squat jumps (see Video 4, Supplemental Digital Content, http://links.lww.com/SCJ/A186 and Figure 3).

## LIMITATIONS

This article does not take into account that the Gmed can be anatomically subdivided into 3 parts, all of which can experience different levels of activity depending on exercise selection (32). Additionally, Gmed activation can vary as a consequence of different strength ratios of the Gmed to the quadriceps or hamstrings (43,45); altering the exercise intensity (44); changing the kinematics (10); changing the way the eccentric actions are performed (21); training experience (8,17,27); and asymmetrical loading (44). Another issue, which has not been included in this article, is the activity ratio between Gmed and the tensor fasciae latae. Thus, exercises with greater tensor fasciae latae activity than Gmed activity, such as forward lunges, should be coupled with other exercises with reversed reciprocal activity that favors the Gmed such as the clam (clamshell), sidestep, unilateral bridge, and quadruped hip extension (40).

## PRACTICAL APPLICATION

This article summarizes the process leading up to a Gmed strengthening program: the identification of Gmed weakness, selection of Gmed exercises, and implementation Gmed exercises into resistance training sessions. Readers can apply handheld strength measurements easily in the gym, select a combination of thirty Gmed exercises from Tables 1 and 2, and apply them to a beginner's or advanced resistance training workout as described in Tables 3–5.

## Figures and Tables

**Figure:**
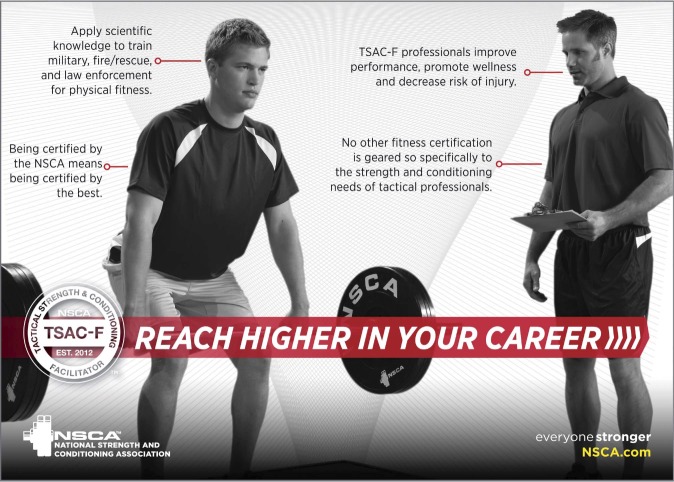
No available caption
